# Supplementary material for: Bichir external gills arise via heterochronic shift that accelerates hyoid arch development
Source: eLife. 2019 Mar 26;8:e43531. doi: 10.7554/eLife.43531 (PMC6440740; doi:10.7554/eLife.43531)
Supplement: Transparent reporting form [file elife-43531-transrepform.docx]

***eLife’s* transparent reporting form**

We encourage authors to provide detailed information *within their submission* to facilitate the interpretation and replication of experiments. Authors can upload supporting documentation to indicate the use of appropriate reporting guidelines for health-related research (see [EQUATOR Network](http://www.equator-network.org/%20)), life science research (see the [BioSharing Information Resource](https://biosharing.org/)), or the [ARRIVE guidelines](http://www.plosbiology.org/article/info:doi/10.1371/journal.pbio.1000412) for reporting work involving animal research. Where applicable, authors should refer to any relevant reporting standards documents in this form.

If you have any questions, please consult our Journal Policies and/or contact us: [editorial@elifesciences.org](mailto:editorial@elifesciences.org).

**Sample-size estimation**

- You should state whether an appropriate sample size was computed when the study was being designed
- You should state the statistical method of sample size computation and any required assumptions
- If no explicit power analysis was used, you should describe how you decided what sample (replicate) size (number) to use

Please outline where this information can be found within the submission (e.g., sections or figure legends), or explain why this information doesn’t apply to your submission:

Sample-size estimation does not apply to the data in the submitted manuscript.

This does not apply to our submission: sample size of descriptive data

**Replicates**

- You should report how often each experiment was performed
- You should include a definition of biological versus technical replication
- The data obtained should be provided and sufficient information should be provided to indicate the number of independent biological and/or technical replicates
- If you encountered any outliers, you should describe how these were handled
- Criteria for exclusion/inclusion of data should be clearly stated
- High-throughput sequence data should be uploaded before submission, with a private link for reviewers provided (these are available from both GEO and ArrayExpress)

Please outline where this information can be found within the submission (e.g., sections or figure legends), or explain why this information doesn’t apply to your submission:

Fate mapping experiments (stated in line 106 of the manuscript) involved the injection of CM-DiI cell tracker into embryonic neural folds and subsequent analysis of the presence of labeled cells within the external gill. In total, out of 21 embryos, 16 displayed presence and 5 absence of CM-DiI in the external gills. This analysis involved biological replicates only.

Pharmacological treatments (stated in lines 154 and 156 of the manuscript) involved the application of Fgf signaling inhibitor SU5402 (dissolved in DMSO), or DMSO at the equivalent concentration. The treated embryos were analysed for the presence/absence of endodermal outpocketing within the hyoid region. Two independent experiments were performed. 17th Mar 2017, DMSO control: out of 8 embryos, 8 showed the presence of endodermal outpocketings, SU5402 treat: out of 5 embryos, 5 showed the absence of endodermal outpocketings. 15th Dec 2017, DMSO control: out of 10 embryos, 10 showed the presence of endodermal outpocketings, SU5402 treat: out of 10 embryos, 9 showed absence and 1 showed the presence of endodermal outpocketings. This analysis involved biological replicates only.

**Statistical reporting**

- Statistical analysis methods should be described and justified
- Raw data should be presented in figures whenever informative to do so (typically when N per group is less than 10)
- For each experiment, you should identify the statistical tests used, exact values of N, definitions of center, methods of multiple test correction, and dispersion and precision measures (e.g., mean, median, SD, SEM, confidence intervals; and, for the major substantive results, a measure of effect size (e.g., Pearson's r, Cohen's d)
- Report exact p-values wherever possible alongside the summary statistics and 95% confidence intervals. These should be reported for all key questions and not only when the p-value is less than 0.05.

Please outline where this information can be found within the submission (e.g., sections or figure legends), or explain why this information doesn’t apply to your submission:

Our results do not contain statistical analyses.

(For large datasets, or papers with a very large number of statistical tests, you may upload a single table file with tests, Ns, etc., with reference to sections in the manuscript.)

**Group allocation**

- Indicate how samples were allocated into experimental groups (in the case of clinical studies, please specify allocation to treatment method); if randomization was used, please also state if restricted randomization was applied
- Indicate if masking was used during group allocation, data collection and/or data analysis

Please outline where this information can be found within the submission (e.g., sections or figure legends), or explain why this information doesn’t apply to your submission:

In the case of fate-mapping experiments and pharmacological treatments, embryos were taken from the same breeding batches (as stated in the “Replicates” section). No selection was done to allocate embryos into either control or experimentally manipulated groups and no exclusion of any embryo was done during the duration or at the end of the experiments.

In case of analysis of wild-type embryos, our samples come from several breeding seasons over the past several years, and the morphological analyses are fully repeatable.

**Additional data files (“source data”)**

- We encourage you to upload relevant additional data files, such as numerical data that are represented as a graph in a figure, or as a summary table
- Where provided, these should be in the most useful format, and they can be uploaded as “Source data” files linked to a main figure or table
- Include model definition files including the full list of parameters used
- Include code used for data analysis (e.g., R, MatLab)
- Avoid stating that data files are “available upon request”

Please indicate the figures or tables for which source data files have been provided:

Does not apply.
